# Development of a nomogram model for the early prediction of sepsis-associated acute kidney injury in critically ill patients

**DOI:** 10.1038/s41598-023-41965-x

**Published:** 2023-09-14

**Authors:** Milin Peng, Fuxing Deng, Desheng Qi

**Affiliations:** 1 Department of Critical Care Medicine, Xiangya Hospital, Central South University, Changsha, Hunan People’s Republic of China; 2 Department of Oncology, Xiangya Hospital, Central South University, Changsha, Hunan People’s Republic of China; 3 Department of Emergency, Xiangya Hospital, Central South University, Xiangya Road 87, Changsha, Hunan People’s Republic of China; 4 National Clinical Research Center for Geriatric Disorders, Xiangya Hospital, Central South University, Changsha, Hunan People’s Republic of China; 5 Hunan Provincial Clinical Research Center for Critical Care Medicine, Xiangya Hospital, Central South University, Changsha, Hunan People’s Republic of China

**Keywords:** Acute kidney injury, Bacterial infection, Risk factors

## Abstract

Sepsis-associated acute kidney injury is a common complication of sepsis, but it is difficult to predict sepsis-associated acute kidney injury. In this retrospective observational study, adult septic patients were recruited from the MIMIC-III database as the training cohort (n = 4764) and from Xiangya Hospital (n = 1568) and Zhang’s database as validation cohorts. We identified eleven predictors with seven independent risk predictors of sepsis-associated acute kidney injury [fluid input_day1 ≥ 3390 ml (HR hazard ratio 1.42), fluid input_day2 ≥ 2734 ml (HR 1.64), platelet_min_day5 ≤ 224.2 × 10^9^/l (HR 0.86), length of ICU stay ≥ 2.5 days (HR 1.24), length of hospital stay ≥ 5.8 days (HR 1.18), Bun_max_day1 ≥ 20 mmol/l (HR 1.20), and mechanical ventilation time ≥ 96 h (HR 1.11)] by multivariate Cox regression analysis, and the eleven predictors were entered into the nomogram. The nomogram model showed a discriminative ability for estimating sepsis-associated acute kidney injury. These results indicated that clinical parameters such as excess input fluid on the first and second days after admission and longer mechanical ventilation time could increase the risk of developing sepsis-associated acute kidney injury. With our study, we built a real-time prediction model for potentially forecasting acute kidney injury in septic patients that can help clinicians make decisions as early as possible to avoid sepsis-associated acute kidney injury.

## Introduction

Sepsis-associated acute kidney injury (SA-AKI) is one of the most common complications in septic patients. SA-AKI has a high incidence rate of greater than 60% in patients with septic shock^1^. Sepsis is the leading cause of AKI in patients^2,3^. AKI contributes to death in sepsis and is an independent risk factor for in-hospital mortality in septic patients^1,4^. Moreover, SA-AKI easily develops into persistent and chronic renal failure^5^, and persistent SA-AKI has a higher risk of in-hospital mortality than transient SA-AKI^6^. Hence, SA-AKI places a heavy economic burden on global health^7,8^.

Furthermore, AKI develops early, within 24 h after the onset of septic shock, and survival is lower for septic shock patients with early AKI^1^. If SA-AKI occurs, patients will have a higher odds ratio of mortality or probability of progressing to chronic kidney failure^5^. Therefore, early detection and intervention of SA-AKI is crucial for providing an opportunity to improve the survival of septic patients and eliminate chronic renal disease transformation^9,10^. Traditional serum creatine and urine output are tests with limitations and time lag features for predicting SA-AKI^5^. The predictive models of the occurrence of SA-AKI developed over time. A prediction model of SA-AKI was established by logistic regression analysis and identified diabetes mellitus, chronic kidney disease, congestive heart failure and other conditions as risk factors for the occurrence of SA-AKI^11^. A mathematical model integrated with antithrombin III is used to forecast SA-AKI^12^. Data from a retrospective study led to the conclusion that a decline in complete C3 and extension of activated partial thromboplastin time (APTT) can predict SA-AKI^13^.

However, the predictive power of methods presented in previous studies is restricted due to a lack of real-time data and a small sample size. In this study, we aimed to develop a clinical prediction model for providing early prediction of the new onset of AKI in septic patients.

## Results

### Baseline characteristics

A total of 5784 patients diagnosed with sepsis 3.0 were collected in the training database according to the inclusion criteria. A total of 1020 patients with chronic renal disease or renal dysfunction were excluded. Among the remaining 4764 patients, 3013 (63.2%) had SA-AKI within 5 days after ICU admission according to Kidney Disease Improving Global Outcome (KIDGO) criteria. A total of 1652 patients diagnosed with sepsis 3.0 from Xiangya Hospital were included as validation cohort I according to the inclusion criteria. Eighty-four patients with chronic renal disease or renal dysfunction were excluded. Among the remaining 1568 patients, 669 (42.7%) had SA-AKI within 5 days after ICU admission according to the KIDGO criteria. We used the database from Zhang’s study^14^ as validation cohort II. In this cohort, we extracted clinical data of patients with sepsis (304 patients) and included 232 patients after screening with inclusion and exclusion criteria. Ninety-seven patients had SA-AKI (41.8%) among the 232 patients with sepsis.

The baseline characteristics and prognostic results of the training cohort and validation cohort I are shown in Table 1. In the training group, sepsis patients were older (65 vs. 59 years), had a longer ventilation time (70.79 vs. 16.68 h), had a higher incidence of chronic lung disease (23% vs. 12%) and diabetes (24% vs. 18%), had lower incidences of males (55% vs. 65%), chronic heart disease (28% vs. 40%), and tumours (12% vs. 18%) and had a higher 180-day mortality (26% vs. 21%) and 365-day mortality (30% vs. 22%) than patients in validation cohort I. Patients in validation cohort I had a longer hospital stay (14.56 vs. 7.24 days, P < 0.001) and ICU stay duration (7 vs. 2.53 days, P < 0.001) than patients in the training cohort. These two cohorts had balanced characteristics of 30-day mortality (18% vs. 20%, P = 0.26) and SOFA score (4% vs. 4%, P = 0.789).

**Table 1:** Baseline clinical characteristics between training group and validation group in patients with sepsis.

Variables	Total (n = 6332)	Training cohort (n = 4764)	Validation cohort (n = 1568)	P
Age, median (IQR)	63.71 (51, 76.55)	65.18 (52.38, 78.51)	59 (47, 70)	< 0.001
Male, n (%)	3629 (57)	2602 (55)	1027 (65)	< 0.001
SOFA, median (Q1, Q3)	4 (3, 7)	4 (3, 6.25)	4 (2, 10)	0.789
Underlying disease, n (%)				
Chronic heart disease	1986 (31)	1353 (28)	633 (40)	< 0.001
Chronic lung disease	1291 (20)	1103 (23)	188 (12)	< 0.001
Tumor	855 (14)	574 (12)	281 (18)	< 0.001
Diabetes	1431 (23)	1143 (24)	288 (18)	< 0.001
Liver disease	591 (9)	450 (9)	141 (9)	0.627
Length of hospital stay, median (Q1, Q3)	8.27 (4.7, 15.49)	7.24 (4.27, 12.52)	14.56 (7.39, 26.58)	< 0.001
Length of ICU stay, median (Q1, Q3)	3.06 (1.66, 7.49)	2.53 (1.42, 5.14)	7 (4, 15)	< 0.001
Ventilation hour, median (Q1, Q3)	70.79 (14.13, 70.79)	70.79 (24, 70.79)	16.68 (0, 113.4)	< 0.001
Mortality, n (%)				
Hospital mortality				
Mortality at day 30	1177 (19)	870 (18)	307 (20)	0.260
Mortality at day 180	1577 (25)	1245 (26)	332 (21)	< 0.001
Mortality at day 365	1748 (28)	1406 (30)	342 (22)	< 0.001

*IQR* interquartile range, *SOFA* sequential organ failure assessment, *AKI* acute kidney injury.

### Independent prognostic factors related to AKI occurrence in septic patients

The characteristics in univariate Cox analysis were used to clarify the potential risk factors associated with SA-AKI in the training group, which are shown in Supplementary Table 1.

We then used the ROC method to explore the predictive efficiency for AKI occurrence in continuous variables with significant differences after univariate Cox regression analysis. As the ROC method showed, the optimal cutoff values of fluid input volume at 24 h and 48 h after ICU admission (fluid input_day1, fluid input_day2), length of ICU stay, length of hospital stay, minimum and maximum levels of urea nitrogen at 24 h after ICU admission (Bun_min_day1, Bun_max_day1), mechanical ventilation time, minimum levels of alanine aminotransferase (ALT) at 24 h after ICU admission (Alt_min_day1), minimum levels of platelets at 24 h after ICU admission (platelet_min_day5), and total fluid output volume from Day 1 to Day 3 after ICU admission (total fluid output_day3) were 3390 ml, 2734 ml, 2.5 days, 5.8 days, 20 mmol/l, 20 mmol/l, 96 h, 93 U/l, 224.2 × 10^9^/l, and 5684 ml, respectively.

In the multivariate Cox regression analysis in the training group, we identified eleven predictors of SA-AKI: fluid input_day1, fluid input_day2, length of ICU stay, length of hospital stay, Bun_min_day1, Bun_max_day1, mechanical ventilation time, Alt_min_day1, platelet_min_day5, and total fluid output_day3. In those predictors, fluid input_day1 ≥ 3390 ml (HR 1.42; 95% CI 1.07–1.87; P = 0.014), fluid input_day2 ≥ 2734 ml (HR 1.64; 95% CI 1.42–1.89; P < 0.001), platelet_min_day5 ≤ 224.2 × 10^9^/l (HR 0.86; 95% CI 0.76–0.97; P = 0.015), length of ICU stay ≥ 2.5 days (HR 1.24; 95% CI 1.14–1.35; P < 0.001), length of hospital stay ≥ 5.8 days (HR 1.18; 95% CI 1.09–1.28; P < 0.001), Bun_max_day1 ≥ 20 mmol/l (HR 1.20; 95% CI 1.11–1.29; P < 0.001), mechanical ventilation time ≥ 96 h (HR 1.11; 95% CI 1.03–1.20; P = 0.008) were the independent risk predictors of SA-AKI (Table 2).

**Table 2:** Multivariate Cox regression analysis obtains independent risk factors impacting on AKI coming up in sepsis.

Variables	Hazard ratio	95% CI	P value
Total fluid input_day1	1.42	1.07–1.87	0.014
Total fluid input_day2	1.64	1.42–1.89	< 0.001
Out_3day_total	0.85	0.56–1.30	0.458
Antibiotic_group5	1.27	0.80–2.02	0.314
Platelet_min_day5	0.86	0.76–0.97	0.015
Bun_min_day1	1.04	0.97–1.12	0.281
Bun_max_day1	1.20	1.11–1.29	< 0.001
ALT_min_day1	0.99	0.92–1.07	0.803
Mechanical ventilation time	1.11	1.03–1.20	0.008
Length of ICU stay	1.24	1.14–1.35	< 0.001
Length of hospital stay	1.18	1.09–1.28	< 0.001

### Nomogram development and validation

**Figure 1:**
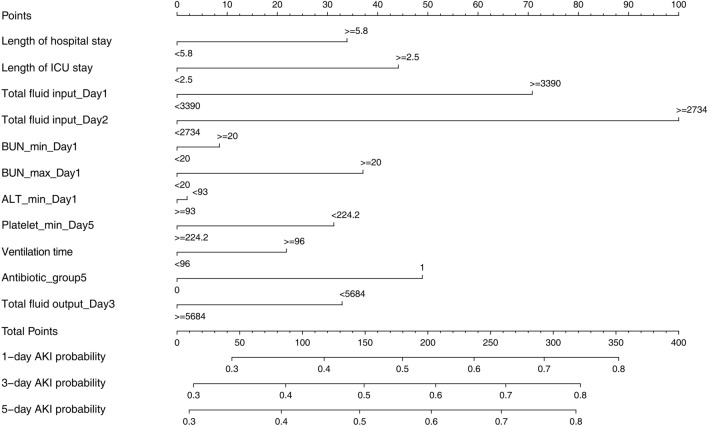
Nomogram prediction model for foretelling sepsis induced acute renal injury. The nomogram model was constructed based on the independent risk factors with respective cutoffs from the multivariate Cox regression analysis of the training cohort. According to the contribution of each influencing factor in the model to the outcome variable (the size of the regression coefficient), each value level of each influencing factor was assigned a score, and then the scores were added to obtain the total score. Finally, through the function transformation relationship between the total score and the probability of the outcome event, the predicted value of the individual outcome event was calculated. *ICU* intensive care units, *BUN* blood urea nitrogen, *ALT* alanine aminotransferase, *AKI* acute kidney injury.

**Figure 2:**
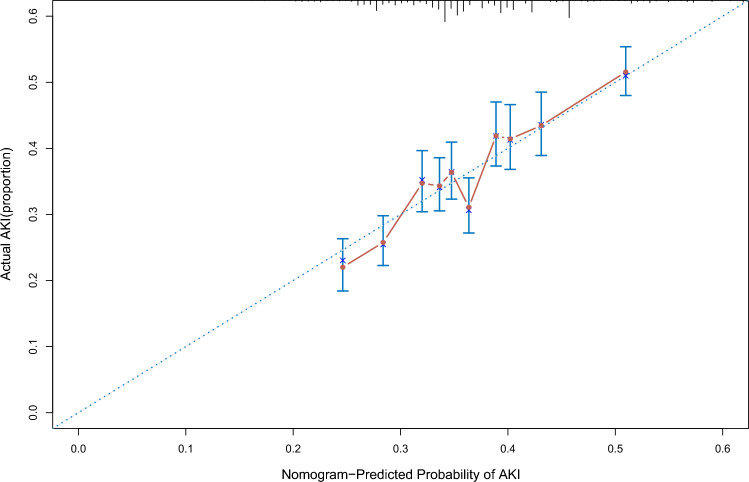
Calibration curve of the nomogram for the training cohort. The x-axis represented the nomogram predicted probability of AKI, and the y-axis showed actual situation of onset of AKI. The calibration plot was used to demonstrate the consistency between predicted probability of AKI occurrence figured out by the nomogram and the actual probability of AKI onset for the training cohort.

**Figure 3:**
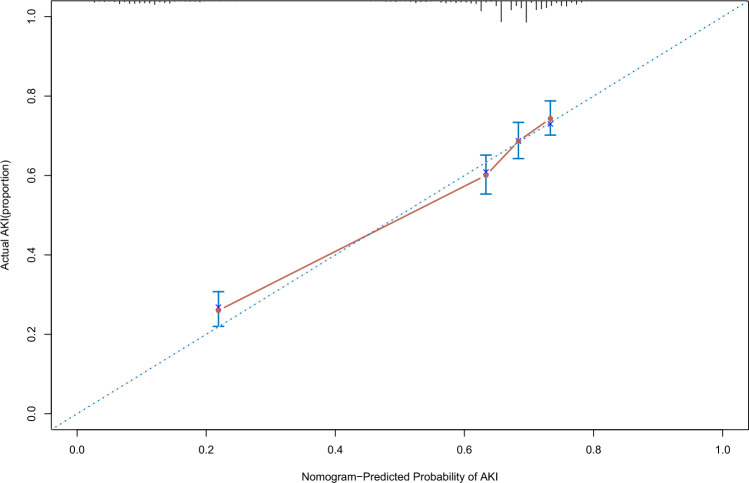
Calibration curve of the nomogram for the validation cohort. The calibration plot was used to exhibit the consistency between predicted probability of AKI occurrence figured out by the nomogram and the actual probability of AKI onset for the validation cohort.

We established a SA-AKI prediction nomogram according to the resulting coefficients from the multivariate Cox analysis in the training cohort from the MIMIC-III database and verified it in validation cohort I from Xiangya Hospital (Fig. 1). This nomogram model had superior predictive power to predict AKI occurrence in sepsis, with a C-index of 0.586 (95% CI 0.570–0.601) in the training cohort and 0.715 (95% CI 0.691–0.739) in validation cohort I. Our nomogram prediction model was also validated by validation cohort II from Zhang’s database^14^, with a C-index of 0.611 (95% CI 0.480–0.741) in this database from China. The C-index of the prognostic model and single risk factors for the training cohort and validation cohort I are shown in Table 3. Calibration curves for the estimation of AKI occurrence revealed excellent consistency between the AKI occurrence estimates from the nomogram and the actual AKI incidence rate based on the KDIGO criteria in the two cohorts (Figs. 2, 3). The nomogram-generated scores for each patient demonstrated the superior performance of the nomogram model to discriminate AKI and non-AKI (Supplementary Figs. 2, 3). With the 11 variables that constructed the nomogram, each covariate in the model was assigned a score by drawing a vertical line vertically downwards to the x-axis. By adding the total score and locating it to a three total point scale through a vertical line, 1-day, 3-day, and 5-day AKI probabilities were determined. Moreover, the DCA curve showed that the nomogram model had outstanding clinical utility performance and was worthy of clinical use (Supplementary Fig. 4).

**Table 3:** C-index of proposed nomogram model and single risk factors of nomogram.

Model	C-index	
	Training cohort	Validation cohort
Prognostic models		
Proposed nomogram	0.59	0.72
Single risk factors		
Total fluid input_day1	0.63	0.51
Total fluid input _day2	0.63	0.50
Bun_max_day1	0.65	0.78
Antibiotic_group5	0.51	0.51
Length of ICU stay	0.72	0.51
Length of hospital stay	0.68	0.52
Bun_min_day1	0.61	0.78
Mechanical ventilation time	0.55	0.52
Alt_min_day1	0.52	0.50
Platelet_min_day5	0.53	0.57
Total fluid output_day3	0.54	0.51

## Discussion

The rapid onset and great harm of SA-AKI necessitates early and precise detection. Determining whether a septic patient has a high risk of developing AKI at a certain time requires a real-time predictive tool to provide an opportunity for timely prevention and treatment. However, there are still controversies about when AKI occurs in the rapidly progressive course of sepsis. In a few studies, scholars found high risks for SA-AKI and built models to predict AKI onset in sepsis, but the models lacked real-time characteristics and produced delayed and obscure prediction results of questionable clinical application value^6,11,12^.

To address this problem, we established a predictive model by using a nomogram to estimate AKI occurrence that included available clinical variables with time tags based on structured big data from the MIMIC-III database. The prognostic model was validated by the Xiangya dataset and Zhang’s database. To our knowledge, this is the first real-time predictive model to predict the new onset of AKI in septic patients. We can obtain respective 1-day, 3-day and 5-day AKI probabilities through the nomogram. In the model, a few clinical variables significantly associated with the occurrence of SA-AKI were screened out by multivariate Cox analysis. Most of these variables were real-time parameters that can instruct clinicians clearly and concisely. We made all the included variables dichotomous according to the cutoff of power to predict SA-AKI for each variable. Hence, the dichotomous and real-time format of clinical parameters enables clinicians to receive clear and urgent instructions that are helpful in making decisions, which is vital in the environment of the emergency department and ICU.

We can obtain useful clinical information from the nomogram. Infusion volume management is critical in the emergency department and ICU. From our nomogram, input fluid volume on the first day after admission to the ICU of ≥ 3390 ml, input fluid volume on the second day of ≥ 2733 ml, or total fluid output in the first three days of < 5683 ml can maximally exacerbate the fluid imbalance in septic patients and lead to AKI. Excessive fluid load can bring a heavy burden to the kidney, heart and lung in septic patients and result in tissue oedema and reduced oxygen delivery to these organs. Cardiac dysfunction can aggravate renal injury^15^. A length of hospital stay of ≥ 5.8 days or length of ICU stay of ≥ 2.5 days adds to the probability of developing SA-AKI according to the nomogram. In a recent study, patients with AKI after noncardiac surgery had a longer length of hospital stay (21 days, 95% CI 14–31, P < 0.001) and longer length of ICU stay (2 days, 95% CI 2–5, P < 0.001) than non-AKI patients^16^. Therefore, our results suggest that control of the length of ICU stay to < 2.5 days and hospital stay to < 5.8 days can decrease the chance of developing SA-AKI. The minimum amount of platelets on the fifth day after admission to the ICU of < 224.2 is associated with a high risk of developing SA-AKI according to the nomogram. Platelets are activated and consumed with the formation of microthrombi and microvascular dysfunction and play an important role in SA-AKI occurrence^17^. A lower platelet count is an independent risk factor for the severity of AKI^18^ and an independent predictor of SA-AKI^19^. In our study, we further determined the exact time and count of decreasing platelets that affect the occurrence of SA-AKI. A longer mechanical ventilation time in the ICU has been known to potentially affect the deterioration of renal function in critically ill patients^20,21^. Our results demonstrate that ventilation time ≥ 96 h is the available cutoff to discriminate the amount of ventilation time that has an effect on SA-AKI occurrence. Vancomycin is a commonly used antibiotic in the ICU and is known to have renal toxicity. Our results show that the use of vancomycin has the potential to lead to SA-AKI occurrence. In summary, our study provides a real-time predictive tool constructed by timely available clinical variables to forecast the occurrence of SA-AKI. The study has several limitations. First, selection bias cannot be avoided due to the respective nature of our study. Nevertheless, we have included a large sample size by using rigorous inclusion criteria for sepsis of the Sepsis 3.0 standard, defining AKI by KDIGO criteria and excluding patients with previous kidney disease. These strict means may reduce the selection bias. Second, the validation cohorts of the Xiangya Hospital dataset and Zhang’s database are unicentral, making our conclusions lacking in generalizability, so a multicentre dataset is needed to further confirm our prognostic model for predicting SA-AKI. Third, we did not discuss the trajectory of SA-AKI in our study, which should be taken into consideration. However, the trajectory of SA-AKI was discussed in detail in Zhang’s outstanding research^22^, which is of great value to us. Fourth, the prediction model integrating clinical parameters and gene/protein signalling networks is very important to sketch the contours and understand the intrinsic nature of SA-AKI in future studies^23,24^. Fifth, computational biology is a very useful methodology and tool for dissecting insights into gene markers and related diseases^24–26^ and would provide a new direction for predicting the emergence of SA-AKI. Sixth, although we used data with time characteristics in our study and incorporated data with time tags in the prediction model, the problem of time-varying covariates was not fully addressed. Zhang’s research probed this area in depth^27^, and we will adopt the advanced methodology of Zhang’s research in future studies.

In conclusion, this research establishes a real-time predictive model for the early occurrence of AKI in septic patients in the ICU. The nomogram model successfully identifies several critical clinical variables that are associated with a high risk of developing early AKI in sepsis. Moreover, the real-time and dichotomous characteristics of these clinical variables and the nomogram may have the potential to give clinicians prompt and clear instructions as early as possible so that timely decisions to avoid SA-AKI can be made.

## Methods

### Research object

We included patients from the MIMIC-III database as the training cohort and patients from Xiangya Hospital as the validation cohort. The research objects of the training cohort were adult critically ill patients ≥ 18 years old diagnosed with sepsis according to the Sepsis 3.0 criteria. The MIMIC-III database was developed and maintained in collaboration with the Massachusetts Institute of Technology (MIT) and Harvard Medical School. It comprises data on ICU patients at Harvard Beth Israel Deaconess Medical Center from June 2001 to October 2012 and contains clinical information on 53,423 adult patients (age 16 and older), totaling more than 300 million structured data entries. The database has undergone rigorous deidentified processing and is freely available to researchers worldwide after coapproval by the ethical review boards of MIT and Harvard Medical School.

The validation cohort I was designed in accordance with the Strengthening the Reporting of Observational Studies in Epidemiology (STROBE) reporting guidelines. We retrospectively collected the clinical information of critically ill adult patients ≥ 18 years old diagnosed with sepsis according to the Sepsis 3.0 criteria^28^ from the electronic medical record system of Xiangya Hospital of Central South University in China from 2016 to 2021. All private clinical information was deidentified. The procedures in this study were performed in accordance with the ethical standards of human experimentation. This study of “Prognostic factors and predictive models for septic patients” was reviewed and approved by the Ethics Committee of Xiangya Hospital of Central South University with ethical code 202111218 on November 17, 2021. The Ethics Committee of Xiangya Hospital of Central South University waived the need to obtain informed consent. The procedures in this study were performed in accordance with the Helsinki Declaration of 1975.

The clinical data of the study subjects in validation cohort II were extracted from Zhang’s database^14^. The study was carried out in Zigong Fourth People’s Hospital, Sichuan, China, from 2019 to 2020. The Ethics Committee of Zigong Fourth People’s Hospital approved this study (Approval Number: 2021-014). Informed consent was waived because of the retrospective design of the study. The study was in line with the Declaration of Helsinki. The study subjects were ≥ 18-year-old ICU patients diagnosed with sepsis according to the Sepsis 3.0 criteria. All the private information of objects was deidentified.

### Inclusion criteria of research objects

The research subjects met the following criteria: (1) all the research objects met the diagnostic criteria of sepsis 3.0^28^, sepsis was defined as life-threatening organ dysfunction caused by a dysregulated host response to infection, and organ dysfunction was represented by an increase in the Sequential Organ Failure Assessment (SOFA) score of 2 points or more; (2) the included patients were at least 18 years old; (3) the length of ICU stay of the included patients was longer than 24 h.

### Exclusion criteria of research subjects

Research subjects were excluded based on the following criteria: (1) patients had a history of chronic renal disease or renal dysfunction before the onset of sepsis that had happened this time in our study; (2) patients who did not meet the diagnostic criteria for sepsis 3.0; (3) the patients were under 18 years of age; (4) patients with a length of ICU stay less than 24 h.

### Definitions used in the study

Definition of SA-AKI: According to the KDIGO criteria^29^, sepsis-associated acute kidney injury (SA-AKI) was defined as a serum creatinine increase ≥ 50% within 7 days or ≥ 0.3 mg/dl within 48 h or urine output < 0.5 ml/kg/h for ≥ 6 h within 5 days after ICU admission. (2) The Sequential Organ Failure Assessment (SOFA) score was the total points arrived at by adding up the score of each system comprising respiration, coagulation, liver, cardiovascular, central nervous system, and renal system.

### Data extraction

The clinical data of Xiangya Hospital were deprivatized and approved by the Ethics Committee of Xiangya Hospital of Central South University (No.: 202111218) before implementation. After unified training, researchers used a unified case report form (CRF) to extract database information. The data included all the clinical data from Day 1 to Day 5 after ICU admission: social demographic characteristics, including age, ethnicity, education level, occupation, vital signs, the occurrence of various prognoses (including time), operation code, diagnosis code, drugs, laboratory measurements, medical imaging diagnostic reports, length of hospital stay, outcome, survival data, etc. Our author became a credentialed user of Zhang’s database after completing the required training and extracted clinical data from this database following the same procedure as that used for the Xiangya dataset.

### Time series data

The patient's clinical data were demonstrated with timing characteristics, and they were judged and extracted according to whether the examination time of a certain clinical test fell into the range of the first day to fifth day of hospitalization. Most of the tests, drugs, treatment methods, diagnoses and other indicators have code information, and ICD-9 diagnostic codes were used for diagnosis and treatment. The creators of the Sepsis 3.0 database used the third international consensus definition of sepsis and septic shock for diagnosis coding: suspected infection and SOFA score ≥ 2^28^. SOFA scores were calculated according to the definition of each indicator: oxygenation index, respiratory support, platelets, bilirubin, mean arterial pressure, dose of vasoactive agents, Glasgow score, creatinine, and 24-h urine output. The diagnostic procedure was compared with the criteria of the Disease Control and Prevention Center of United States, Angus, Martin, and ICD-9 codes to confirm the accuracy of the Sepsis 3.0 criteria^30^.

### Data preprocessing

#### Handling missing values

For continuous variables other than age, we used the mean of the corresponding column to fill in missing values. For age data, since the specific age data of patients over 90 years old are missing in the database, we used the normal distribution fitting method to randomly fit the missing age data, assuming that the age of patients with missing age was distributed in the range of 88–99, with an average age of 91.4 years and a standard deviation of 2.

#### Feature engineering of data

The characteristics of the basic diseases were binned and classified. Twelve types of underlying diseases were included in the original database, including congestive heart failure, arrhythmia, hypertension, pulmonary vascular disease, chronic lung disease, lymphoma, metastatic tumour, solid tumour, diabetes (with complications), diabetes (without complications), liver disease, rheumatoid arthritis, etc. We grouped them into five categories: heart-related diseases (congestive heart failure, arrhythmia, hypertension); pulmonary-related diseases (pulmonary vascular disease, chronic lung disease); tumour diseases (lymphoma, metastases, solid tumours); diabetes mellitus (diabetes with comorbidities); and liver disease. We then counted the number of patients with these 5 major categories of disease and included them as variables in the original model.

#### Infection sites and bacterial culture results

We counted the bacterial infection sites of patients on admission and classified them into four categories: no bacterial infection, one bacterial infection, two bacterial infections and more than two bacterial infections. We summarized the bacterial culture results and classified them into two categories: single bacterial infection and multiple bacterial infection, which were included as variables in the original model.

#### Antibiotic use

The 52 antibiotics were classified into 9 categories, including antibiotic Group 1—penicillins/cephalosporins; antibiotic Group 2—the quinolones; antibiotic Group 3—macrolides; antibiotic Group 4—sulfonamides; antibiotic Group 5—vancomycin; antibiotic Group 6—metronidazole; antibiotic Group 7—tetracyclines; antibiotic Group 8—aminoglycoside antibiotics; and antibiotic Group 9—others (rifampin, furantoin, clindamycin). We counted the types and total durations of antibiotics used by patients and included them as variables in the original model.

### Statistical analysis

R software version 4.1.2 (R Project for Statistical Computing) was used for all statistical analyses. Continuous variables are reported as medians and interquartile ranges, and nonnormally distributed data were confirmed by the Kruskal–Wallis test. Continuous variables were compared using the nonparametric Mann‒Whitney test.

The χ^2^ test or Fisher’s exact test was used to compare the differences in categorical variables in the training and validation cohorts. Univariate and multivariate Cox proportional hazard regression analyses were used to screen the independent risk variables, and hazard ratios (HRs) and 95% confidence intervals (CIs) were calculated. A receiver operating characteristic (ROC) cutoff was performed to calculate the cutoff for predicting SA-AKI of each continuous variable with significant differences after univariate logistic regression analysis.

A nomogram was used to establish a visual predictive model on the basis of the independent risk factors according to multivariate regression analysis. The C-index was calculated to judge the discrimination power of the nomogram. A calibration plot was used to depict the consistency between the actual probability of outcome and the predicted probability of the outcome determined by the nomogram. Moreover, the plot of the nomogram-defined score was used to demonstrate the discrimination power of the proposed model. A decision curve analysis (DCA) graph was generated to evaluate the clinical effectiveness of the model.

### Ethics approval and consent to participate

The procedures in this study were accordance with the Helsinki Declaration of 1975 and ethical standards of human experimentation. This study of “Prognostic factors and predictive models for septic patients” was approved by Ethics Committee of Xiangya Hospital of Central South University with the ethical code 202111218 at November 17, 2021. As this was a retrospective observational study, informed consent was waived by the Ethics Committee of Xiangya Hospital of Central South University.

### Supplementary Information

Supplementary Information.

## Data Availability

Raw data supporting the conclusions of this study will be made available by the authors without undue reservation. Correspondence and requests for materials should be addressed to Qi DS.

## Abbreviations

HR: Hazard ratio
KDIGO: Kidney Disease Improving Global Outcome
IQR: Interquartile range
SOFA: Sequential organ failure assessment
AKI: Acute kidney injury
PT: Prothrombin time
PTT: Partial thromboplastin time
WBC: White blood cell
ALT: Alanine aminotransferase
ICU: Intensive care unit
SA-AKI: Sepsis associated acute kidney injury
