# Supplementary material for: Development of a nomogram model for the early prediction of sepsis-associated acute kidney injury in critically ill patients
Source: Sci Rep. 2023 Sep 14;13:15200. doi: 10.1038/s41598-023-41965-x (PMC10502039; doi:10.1038/s41598-023-41965-x)
Supplement: Supplementary file 1 — Supplementary Information. [file 41598_2023_41965_MOESM1_ESM.doc]

**Supplemental Content**

Milin Peng, Fuxing Deng, Desheng Qi. Development of a nomogram model for the early prediction of sepsis-associated acute kidney injury in critically ill patients.

Supplemental graphs:

Supplemental Figure 1: Flow chart for the whole study.

Supplemental Figure 2: Nomogram defined score for the training cohort. In the plot of nomogram defined score, the green vertical lines represented the cases of AKI not happening and red vertical lines represented the cases of AKI onset. The plot of nomogram defined score was used for demonstrating the discrimination power between AKI and non-AKI of the nomogram model in the training cohort.

Supplemental Figure 3: Nomogram defined score for the validation cohort. The plot of nomogram defined score was used for exhibiting the discrimination power between AKI and non-AKI of the nomogram model in the validation cohort.

Supplemental Figure 4: Decision curve analysis graph for the nomogram. The x-axis represented the threshold probability of AKI and the y-axis showed the clinical net benefit. The blue curve was ROC of using the nomogram model, the red curve represented ROC of treating all cases as AKI and the green curve as treating all cases as non-AKI. DCA plot was generated to evaluate the clinical effectiveness of the nomogram model.

Supplemental Table 1: Univariate Cox analysis derived clinical variables influencing AKI occurrence in sepsis. Univariate Cox analysis was used to explore the potential risk factors related with new onset of SA-AKI in the training group. Clinical variables were displayed with time characteristic. Hazard ratios (HR) and 95% confidence interval (CI) were calculated.

This supplemental material has been provided by the authors to give readers additional information about their work.

**Supplemental Figure 1**: The flow chart for the whole study


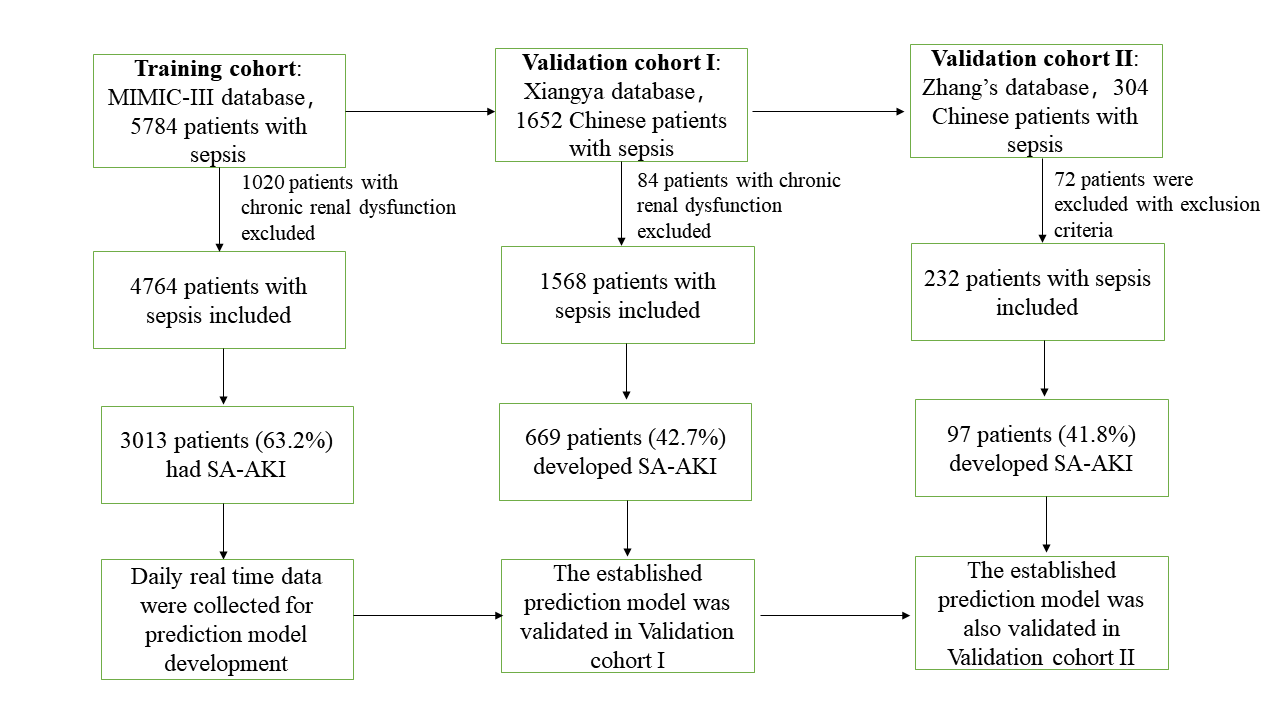


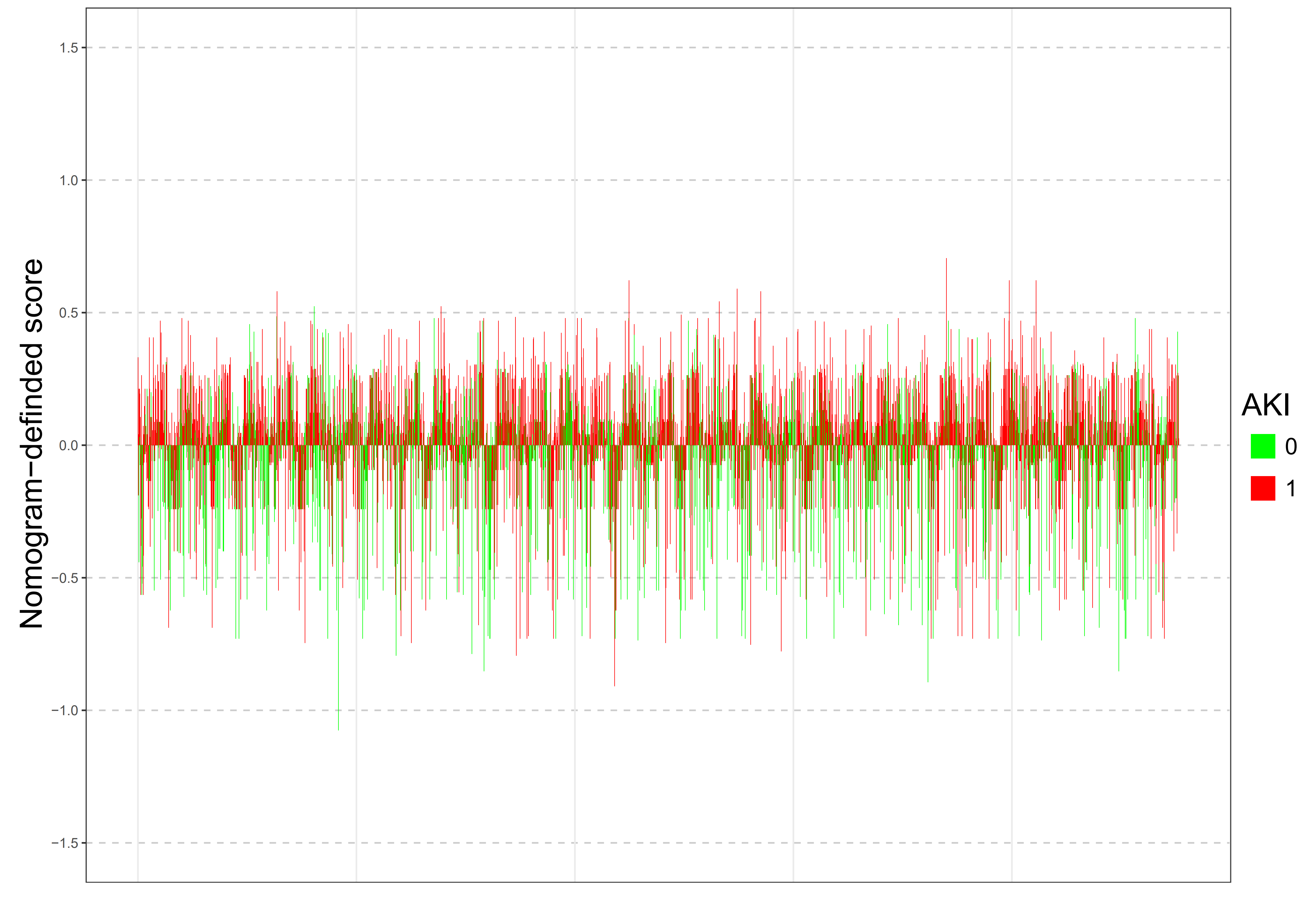
**Supplemental Figure 2**: Nomogram defined score for the training cohort

**Supplemental Figure 3**: Nomogram defined score for the validation cohort


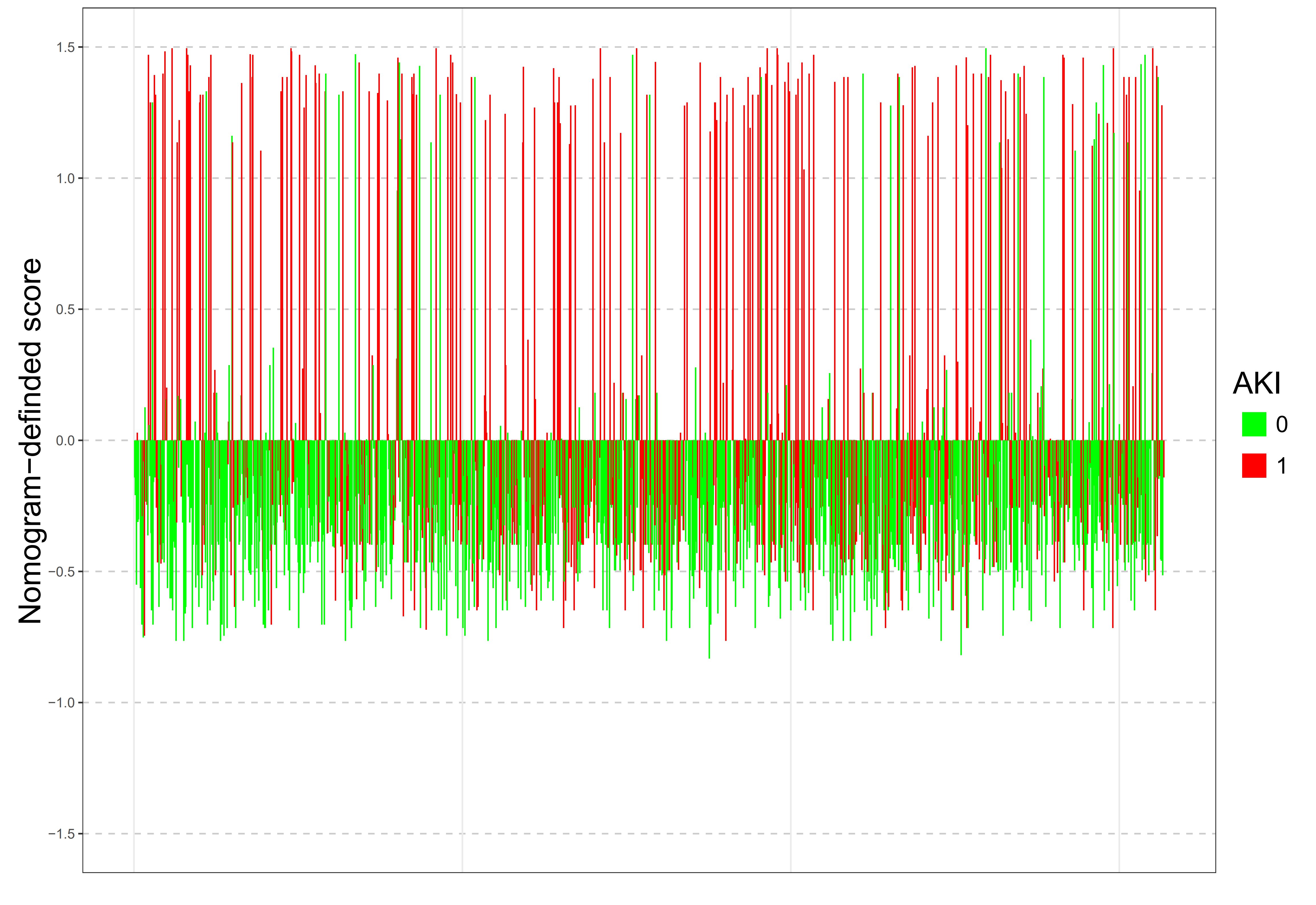


**Supplemental Figure 4**: Decision curve analysis graph for the nomogram


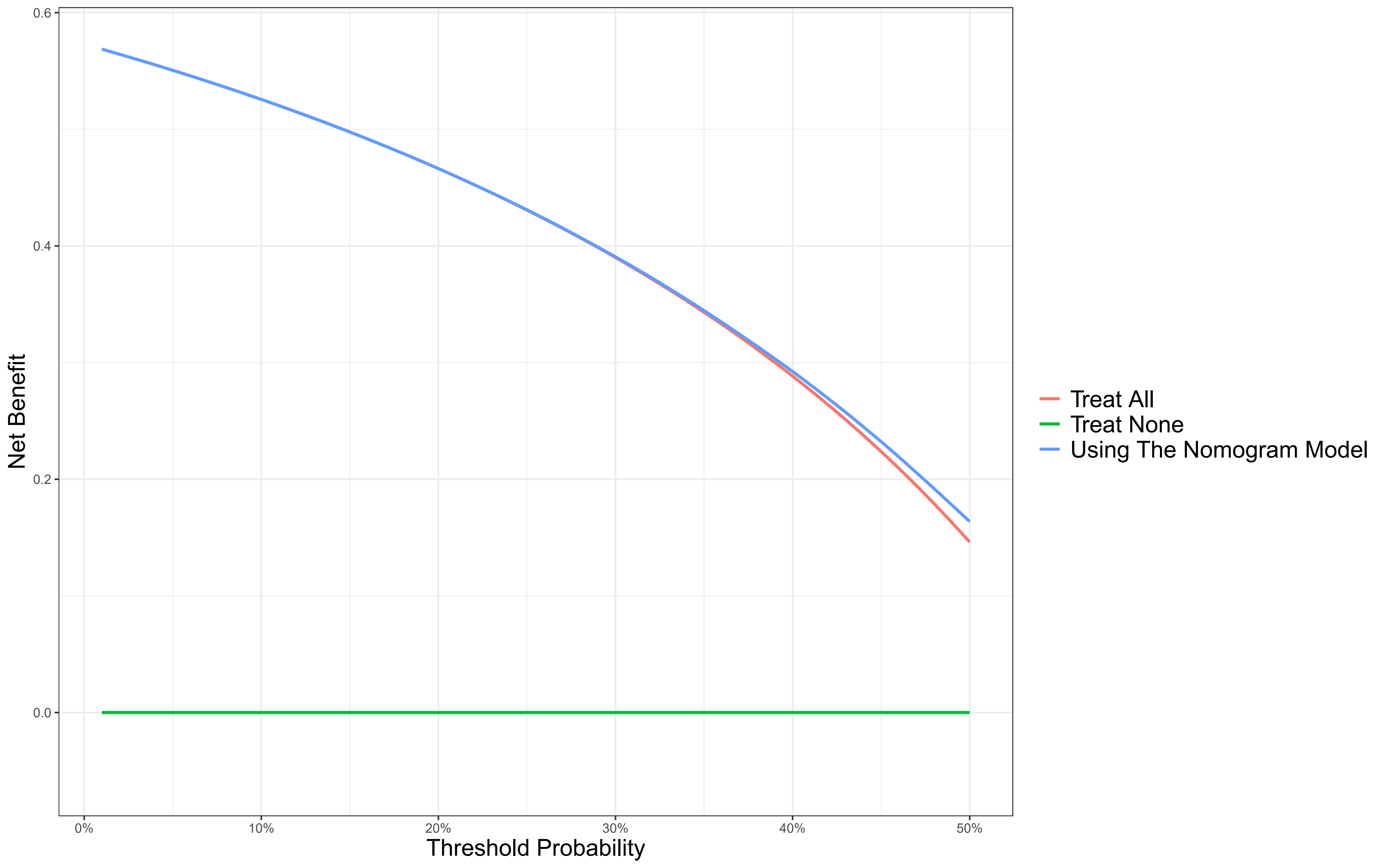


| **Supplemental Table 1**. Univariate Cox analysis derived clinical variables influencing AKI occurrence in sepsis | | | |
| --- | --- | --- | --- |
| **Variables** | **Hazard Ratio** | **95% CI** | **P Value** |
| Sex | 0.98 | 0.87-1.11 | 0.791 |
| Age | 1.04 | 0.96-1.11 | 0.363 |
| Height | 0.91 | 0.80-1.03 | 0.130 |
| Weight | 1.04 | 0.96-1.12 | 0.309 |
| SOFA | 0.99 | 0.91-1.07 | 0.779 |
| Underlying disease | | | |
| Congestive heart failure | 1.25 | 1.01-1.55 | 0.044 |
| Hypertension | 1.14 | 0.63-2.07 | 0.659 |
| Diabetes with complication | 1.51 | 1.04-2.21 | 0.032 |
| Liver disease | 1.09 | 0.83-1.41 | 0.543 |
| Liquid intake and output volume |  |  |  |
| Total fluid input_day1 | 1.44 | 1.09-1.89 | 0.011 |
| Total fluid input__day2 | 1.69 | 1.46-1.95 | <0.001 |
| Total fluid input__day3 | 1.06 | 0.92-1.22 | 0.398 |
| Input_3day_total | 1.50 | 0.87-2.58 | 0.145 |
| Total fluid output_day1 | 0.86 | 0.71-1.04 | 0.129 |
| Total fluid output _day2 | 0.86 | 0.72-1.03 | 0.096 |
| Total fluid output _day3 | 0.84 | 0.69-1.03 | 0.088 |
| Out_3day_total | 0.82 | 0.54-1.25 | 0.353 |
| Antibiotic_group1 | 1.23 | 0.88-1.71 | 0.229 |
| Antibiotic_group2 | 1.07 | 0.88-1.31 | 0.494 |
| Antibiotic_group3 | 1.09 | 0.78-1.52 | 0.602 |
| Antibiotic_group4 | 1.07 | 0.73-1.57 | 0.714 |
| Antibiotic_group5 | 1.23 | 0.78-1.96 | 0.372 |
| Antibiotic_group6 | 0.81 | 0.56-1.18 | 0.268 |
| Antibiotic_group7 | 0.97 | 0.43-2.16 | 0.936 |
| Antibiotic_group8 | 0.53 | 0.07-3.73 | 0.520 |
| Antibiotic_group9 | 1.54 | 0.73-3.22 | 0.257 |
| Laboratory Examination | | | |
| Bicarbonate_min_day1 | 0.93 | 0.87-1 | 0.058 |
| Bicarbonate_max_day1 | 0.99 | 0.92-1.06 | 0.728 |
| Bilirubin_min_day1 | 0.95 | 0.86-1.05 | 0.339 |
| Bilirubin_max_day1 | 0.94 | 0.85-1.04 | 0.254 |
| Creatinine_min_day1 | 1.20 | 1.06-1.35 | 0.004 |
| Creatinine_max_day1 | 1.39 | 1.26-1.54 | <0.001 |
| Hemoglobin_min_day1 | 1.03 | 0.95-1.11 | 0.498 |
| Hemoglobin_max_day1 | 0.93 | 0.87-1.01 | 0.074 |
| Lactate_min_day1 | 1.03 | 0.94-1.13 | 0.543 |
| Lactate_max_day1 | 1.05 | 0.96-1.14 | 0.297 |
| Platelet_min_day1 | 0.98 | 0.88-1.09 | 0.752 |
| Platelet_max_day1 | 1.04 | 0.92-1.19 | 0.514 |
| PT_min_day1 | 1.00 | 0.93-1.07 | 0.977 |
| PT_max_day1 | 0.98 | 0.91-1.05 | 0.559 |
| PTT_min_day1 | 1.05 | 0.98-1.13 | 0.187 |
| PTT_max_day1 | 1.09 | 1.02-1.17 | 0.018 |
| Bun_min_day1 | 1.11 | 1.04-1.20 | 0.003 |
| Bun_max_day1 | 1.15 | 1.07-1.23 | <0.001 |
| WBC_min_day1 | 1.04 | 0.96-1.12 | 0.347 |
| WBC_max_day1 | 1.03 | 0.95-1.10 | 0.480 |
| ALT_min_day1 | 0.95 | 0.88-1.03 | 0.231 |
| ALT_max_day1 | 0.96 | 0.88-1.03 | 0.264 |
| Bicarbonate_min_Day2 | 1.03 | 0.96-1.11 | 0.419 |
| Bicarbonate_max_Day2 | 1.02 | 0.95-1.09 | 0.636 |
| Hemoglobin_min_Day2 | 1.02 | 0.95-1.10 | 0.610 |
| Hemoglobin_max_Day2 | 0.94 | 0.87-1.01 | 0.108 |
| Platelet_min_Day2 | 0.89 | 0.80-0.99 | 0.037 |
| Platelet_max_Day2 | 0.96 | 0.86-1.07 | 0.485 |
| PTT_min_Day2 | 0.95 | 0.89-1.03 | 0.206 |
| PTT_max_Day2 | 0.98 | 0.91-1.05 | 0.510 |
| PT_min_Day2 | 0.95 | 0.88-1.02 | 0.130 |
| PT_max_Day2 | 0.99 | 0.92-1.06 | 0.690 |
| Bun_min_Day2 | 1.11 | 1.03-1.19 | 0.006 |
| Bun_max_Day2 | 1.10 | 1.03-1.19 | 0.007 |
| WBC_min_Day2 | 0.99 | 0.92-1.06 | 0.728 |
| WBC_max_Day2 | 1.05 | 0.97-1.13 | 0.202 |
| Bicarbonate_min_day3 | 0.92 | 0.86-0.99 | 0.023 |
| Bicarbonate_max_day3 | 0.98 | 0.91-1.05 | 0.520 |
| Hemoglobin_min_day3 | 0.95 | 0.88-1.03 | 0.234 |
| Hemoglobin_max_day3 | 1.10 | 1.02-1.19 | 0.013 |
| Platelet_min_day3 | 0.92 | 0.83-1.03 | 0.154 |
| Platelet_max_day3 | 0.89 | 0.80-1.00 | 0.040 |
| Bun_min_day3 | 1.13 | 1.05-1.22 | 0.001 |
| Bun_max_day3 | 1.04 | 0.96-1.11 | 0.341 |
| WBC_min_day3 | 1.01 | 0.93-1.09 | 0.873 |
| WBC_max_day3 | 0.99 | 0.92-1.07 | 0.766 |
| Bicarbonate_min_Day4 | 0.92 | 0.86-0.99 | 0.022 |
| Bicarbonate_max_Day4 | 1.01 | 0.94-1.08 | 0.840 |
| Hemoglobin_min_Day4 | 1.00 | 0.90-1.08 | 0.963 |
| Hemoglobin_max_Day4 | 0.95 | 0.88-1.02 | 0.152 |
| Platelet_min_Day4 | 0.96 | 0.86-1.08 | 0.537 |
| Platelet_max_Day4 | 0.93 | 0.83-1.05 | 0.265 |
| Bun_min_Day4 | 1.06 | 0.99-1.14 | 0.112 |
| Bun_max_Day4 | 1.06 | 0.99-1.14 | 0.086 |
| WBC_min_Day4 | 1.06 | 0.98-1.14 | 0.143 |
| WBC_max_Day4 | 1.05 | 0.97-1.13 | 0.195 |
| Bicarbonate_min_Day5 | 1.01 | 0.94-1.08 | 0.823 |
| Bicarbonate_max_Day5 | 1.01 | 0.94-1.08 | 0.865 |
| Hemoglobin_min_Day5 | 0.98 | 0.91-1.06 | 0.629 |
| Hemoglobin_max_Day5 | 0.92 | 0.86-1.00 | 0.040 |
| Platelet_min_Day5 | 0.84 | 0.74-0.95 | 0.004 |
| Platelet_max_Day5 | 0.86 | 0.76-0.98 | 0.019 |
| Bun_min_Day5 | 0.97 | 0.90-1.04 | 0.416 |
| Bun_max_Day5 | 1.04 | 0.96-1.11 | 0.343 |
| WBC_min_Day5 | 1.09 | 1.01-1.18 | 0.024 |
| WBC_max_Day5 | 1.00 | 0.93-1.08 | 0.938 |
| Mechanical ventilation time | 1.16 | 1.08-1.25 | <0.001 |
| Length of hospital stay | 1.12 | 1.04-1.21 | 0.003 |
| Length of ICU stay | 1.25 | 1.15-1.36 | <0.001 |

PT, prothrombin time; PTT, partial thromboplastin time; WBC, white blood cell; ALT, alanine aminotransferase; ICU, intensive care unit.
